# Towards a systems biology approach to mammalian cell cycle: modeling the entrance into S phase of quiescent fibroblasts after serum stimulation

**DOI:** 10.1186/1471-2105-10-S12-S16

**Published:** 2009-10-15

**Authors:** Roberta Alfieri, Matteo Barberis, Ferdinando Chiaradonna, Daniela Gaglio, Luciano Milanesi, Marco Vanoni, Edda Klipp, Lilia Alberghina

**Affiliations:** 1 Institute for Biomedical Technology – CNR, Via Fratelli Cervi 93, 20090 Segrate, Milan, Italy; 2 Computational Systems Biology, Max Planck Institute for Molecular Genetics, Ihnestraâe 73, 14195 Berlin, Germany; 3 Institute for Biology, Theoretical Biophysics, Humboldt University Berlin, Invalidenstraâe 42, 10115 Berlin, Germany; 4 Department of Biotechnology and Biosciences, University of Milano-Bicocca, Piazza della Scienza 2, 20126 Milan, Italy

## Abstract

**Background:**

The cell cycle is a complex process that allows eukaryotic cells to replicate chromosomal DNA and partition it into two daughter cells. A relevant regulatory step is in the G_0_/G_1 _phase, a point called the restriction (R) point where intracellular and extracellular signals are monitored and integrated.

Subcellular localization of cell cycle proteins is increasingly recognized as a major factor that regulates cell cycle transitions. Nevertheless, current mathematical models of the G_1_/S networks of mammalian cells do not consider this aspect. Hence, there is a need for a computational model that incorporates this regulatory aspect that has a relevant role in cancer, since altered localization of key cell cycle players, notably of inhibitors of cyclin-dependent kinases, has been reported to occur in neoplastic cells and to be linked to cancer aggressiveness.

**Results:**

The network of the model components involved in the G_1 _to S transition process was identified through a literature and web-based data mining and the corresponding wiring diagram of the G_1 _to S transition drawn with Cell Designer notation. The model has been implemented in Mathematica using Ordinary Differential Equations. Time-courses of level and of sub-cellular localization of key cell cycle players in mouse fibroblasts re-entering the cell cycle after serum starvation/re-feeding have been used to constrain network design and parameter determination. The model allows to recapitulate events from growth factor stimulation to the onset of S phase. The R point estimated by simulation is consistent with the R point experimentally determined.

**Conclusion:**

The major element of novelty of our model of the G_1 _to S transition is the explicit modeling of cytoplasmic/nuclear shuttling of cyclins, cyclin-dependent kinases, their inhibitor and complexes. Sensitivity analysis of the network performance newly reveals that the biological effect brought about by Cki overexpression is strictly dependent on whether the Cki is promoting nuclear translocation of cyclin/Cdk containing complexes.

## Background

During the life cycle of eukaryotic cells, DNA replication is restricted to a specific time window, the S phase. Several control mechanisms ensure that each chromosomal DNA sequence is replicated once, and only once, in the period from one cell division to the next. Following S phase, replicated chromosomes separate during mitosis (M phase) and segregate in two nuclei that are then endowed to two newborn cells at division. Two gap phases, called G_1 _and G_2_, separate cell birth from S phase and S phase from M phase, respectively.

When depleted of growth factors, mammalian cells leave G_1 _to enter a reversible quiescent state, referred to as G_0 _[1,2]. Upon growth factor refeeding, signal transduction pathways are activated, ultimately leading to S phase onset. A major control point in the G_0_/G_1 _to S transition has been first identified by Pardee [3], who called it the restriction (R) point. It is defined as the point of the cell cycle in G_1_, after which a cell can enter S phase after removal of growth factors. It occurs at a specific time in G_1 _after re-addition of growth factors, before initiation of S phase. Quiescent cells, before reaching the R point, need continual feeding of nutrients, mitogens and survival factors; in contrast, past the R point, they are irrevocably committed to divide independently from the continuous presence of growth factors in the medium [4]. A control point responding to nutrient availability but with otherwise similar properties, exists also in lower eukaryotes, such as the budding yeast, where it has been named Start [5].

The restriction point R operates stringently in normal cells, but it is defective in cancer cells that accumulate mutations resulting in constitutive mitogenic signaling and defective responses to anti-mitogenic signals that contribute to unscheduled proliferation [6,7]. Mutations that affect the execution of the restriction point mainly occur in two classes of genes: proto-oncogenes and tumor suppressor genes [8]. In normal cells, the products of proto-oncogenes act at different levels along the signaling and regulatory pathways that stimulate cell proliferation. Mutated versions of proto-oncogenes are able to promote tumor growth. Of the more than 100 proto-oncogenes and tumor suppressor genes that have been identified, most function in signal transduction to mimic effects of persistent mitogenic stimulation, thereby uncoupling cells from environmental cues [9]. Their signaling pathways converge on the cycle machinery controlling the passage through the G_1 _phase, by inducing G_1 _cyclins and overriding Cdk inhibitors, preventing cell cycle exit, and ultimately perturbing checkpoint controls [8,10,11]. In the wealth of known oncogenes, many findings indicate that pathways controlled by two tumor suppressor genes, Rb and p53, have been found to be the most frequently disrupted in cancer cells [9,12,13]. Indeed, inactivation of these two tumor suppressor genes results in dysfunction of proteins that normally inhibit cell cycle progression, resulting in either continued proliferation or unscheduled re-entry into the cell cycle, two properties characteristic of most cancer cells [6]. Also the nucleo/cytoplasmic localization of key cell cycle players is relevant: for instance, enhanced cancer aggressiveness has been found to be linked to a preferential cytoplasmic localization of the Cdk inhibitor p27^Kip1 ^[14-18].

The aim of the present report has been to construct a mathematical model of the molecular events that bring a normal quiescent mammalian cell to overcome the restriction point and to enter into S phase. The idea that the logic of cell cycle control is substantially conserved from yeast to mammalian cells is widely accepted [19,20]. Besides, considering relevant the nucleo/cytoplasmic localization of cell cycle players, that is not taken into focus even in recent mathematical models of the G_1_ to S transition in mammalian cells [20-25], we gave specific attention to this aspect in modeling the dynamics of S phase entrance. By using as a framework the model of the G_1 _to S transition in budding yeast developed in our laboratories [26], we present here a computational network model of the dynamics of entrance into S phase of quiescent, normal mammalian cells that is based on extensive data-mining (described in the appendix) and constrained by accompanying experimental data obtained on murine fibroblasts synchronized by serum starvation and stimulated by serum addition to enter S phase. The model allows us to identify the molecular mechanism that underlies the R point, yields specific predictions and gives new insights on the role that the availability of inhibitors of cyclin-dependent kinases (Cki) may have on the entrance into S phase.

## Results

### Building a mathematical model of the G_1 _to S transition network for mammalian cells

**Figure 1:**
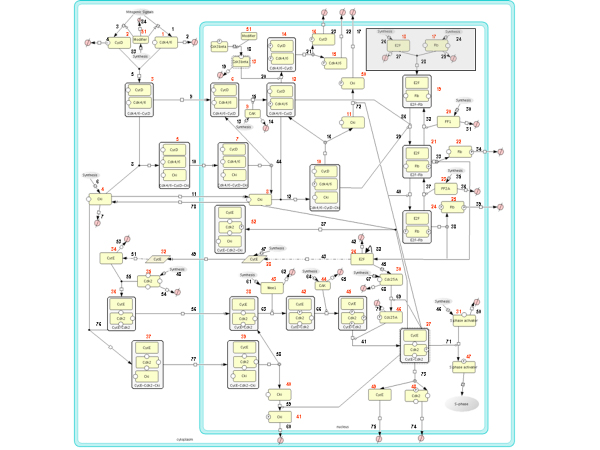
**Processes Regulating the G_1_/S transition in mammalian cells**. Scheme of the G_1 _to S transition of the mammalian cell cycle drawn with Cell Designer. Two compartments are considered, cytoplasm and nucleus. The scheme follows the systems biology graphical notation (SBGN); each component is associated with a red number and each reaction is associated with a black number (Additional file 1). In the gray box a set of reactions which are not explicitly considered for the model.

The data-mining, conducted in order to construct the network of the molecular events that characterize the transition from quiescence into S phase, is described in detail in the Appendix. The relevant players of the network that we considered are: two cyclin/Cdk complexes, i.e. cyclin D1/Cdk4,6 and cyclin E/Cdk2; one Cki inhibitor; the transcription factor E2F and its inhibitor Rb. The nucleo/cytoplasmic localization and the regulatory kinases/phosphatases of this pathway were also taken into account. The following classes of events have been considered: (1) production and degradation of mRNAs and proteins; (2) formation of dimeric and trimeric protein complexes; (3) nucleo/cytoplasmic localization of the compounds, transport processes being described like reactions, (e.g. converting Cdk4cyt into Cdk4nuc); (4) cell growth in terms of volume increase; and hence (5) concentration changes in the nuclear and cytoplasmic compartments. The resulting network was drawn using the notation of Cell designer [27-29] (Figure 1) and the corresponding ODE-based mathematical model was derived (Additional file 1). It describes the dynamics of how different molecular species interact with each other and how they shuttle between cytoplasmic and nuclear compartments.

In essence, the time courses of the network can be summarized as follows (see Appendix for details and references). The first relevant events involve the interplay between the growth-dependent cyclin D, and a generic Cki whose properties more closely resemble those of p27^Kip1^, that has been directly linked to cancer [30], while the role of p21^Cip1 ^in cell cycle was not considered. Cki is endowed to each newborn cell at the end of the previous cycle and plays a dual role, being involved both in inhibition of activity – and promotion of translocation – of cyclin D1/Cdk4,6 and of cyclin E/Cdk2 complexes. In keeping with the notion that Cki-deleted cells are able to translocate Cdk-containing complexes in the nucleus, both complexes are able to translocate in the nucleus either in the Cki-bound or unbound-form. As cyclin D1 builds-up following addition of growth factors to quiescent cells, the cyclin D1/Cdk4,6 complex moves to the nucleus, either alone or bound to Cki. Upon phosphorylation of Rb by nuclear, Cak-phosphorylated cyclin D1/Cdk4,6, the E2F/Rb complex starts to dissociate and E2F-dependent productions of Cdc25A, cyclin E and of more E2F free begins. For simplicity, we assume that all newly-made Cdc25A and E2F will move into the nucleus. Hence, nuclear production rates of these proteins (and of all proteins that appear to be made in the nucleus) sum-up transcription, mRNA export to the cytoplasm, translation and nuclear translocation of the mature protein. Only for cyclin E synthesis and export refer to mRNA production, and a separate translation step is modeled in the cytoplasm.

As cyclin E synthesis proceeds, the cyclin E/Cdk2 complex builds-up and goes to the nucleus, alone or assisted by Cki. Nuclear cyclin E/Cdk2 is sequentially phosphorylated by Wee1 and Cak. After activation by Cdc25A, that removes the Wee1-catalyzed inhibitory phosphorylation, cyclin E/Cdk2 completes Rb phosphorylation. cyclin E/Cdk2 then is taken to phosphorylate a generic activator of the onset of DNA replication (S phase activator for short) that triggers initiation of DNA replication.

### An experimental analysis of the entrance into S phase of quiescent mammalian cells

**Figure 2:**
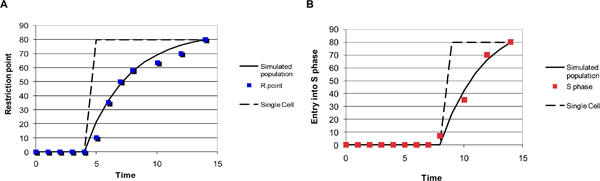
**Temporal parameters of the G_1 _to S transition in resting mammalian fibroblasts stimulated to proliferate by serum**. NIH3T3 cells, made quiescient by serum starvation, were stimulated with 10% serum. For restriction point determination (panel **A**) cells were serum-starved again after different time periods and the fraction of BrdU-positive cells determined 14 hours after serum stimulation. Values of BrdU-positive cells at the end of the 14 hours (total time) are plotted as a function of the simulation time in the presence of serum. Fraction of BrdU-positive cells (**B**) was determined at different time point after serum stimulation. Experimental points are shown by colored symbols. Restriction point overcoming (panel **A**) or S phase state (panel **B**) are shown as on/off binary states by the dashed lines. Data for the population assuming a half-life of growth factor/cell interaction of 2 hours are shown as solid black lines fitting experimental data.

In order to have available an experimental set of data useful to constrain the parameter estimation of the mathematical model described in Figure 1 and in Additional file 1, NIH3T3 murine fibroblasts, brought to quiescence by a 24 hours serum deprivation and stimulated by serum addition for another 14 hours, were analyzed. In the cell population the overcoming of the R point starts at 5 hours and is almost completed at 10 hours (Figure 2A, blue squares), while the entrance into S phase starts at 8–9 hours and is completed at 14 hours (Figure 2B, red squares), following the pattern described in literature [30]. The heterogeneity with which cells overcome the R point and enter into S phase is most likely due to the limiting concentration of "competence" factors, like PDGF [31,32], which are required to rescue the cells from the quiescent state stimulating them to grow and to activate the pathways needed to resume proliferation [33]. Adding 10% serum, the concentration of "competence" factors is limiting: therefore, the interaction of growth factors with their cognate cellular receptors follows a first order kinetics [34]. Enough exposition to the competence factors contained in the serum allows a cell to overcome the R point and to enter into S phase a fixed time after execution of the R point. In order to fit experimental data we must remember that while each individual cell shows a switch-like response for R point overcoming and entrance into S phase (Figure 2A and 2B, dashed lines), cells in a population interact with growth factors according to a first-order kinetics. Assuming a half-life of 2 hrs (i.e. counting that 50% of cells have interacted at two hours, 75% at four hours and so on), satisfactory fitting (Figure 2A and 2B, solid lines) can be obtained for experimental data (Figure 2A and 2B, colored squares). This analysis indicates that overcoming of the R point at the individual cell level takes place after 5 hrs in the presence of the competence factors present in serum and that 4 hrs later S phase starts.

**Figure 3:**
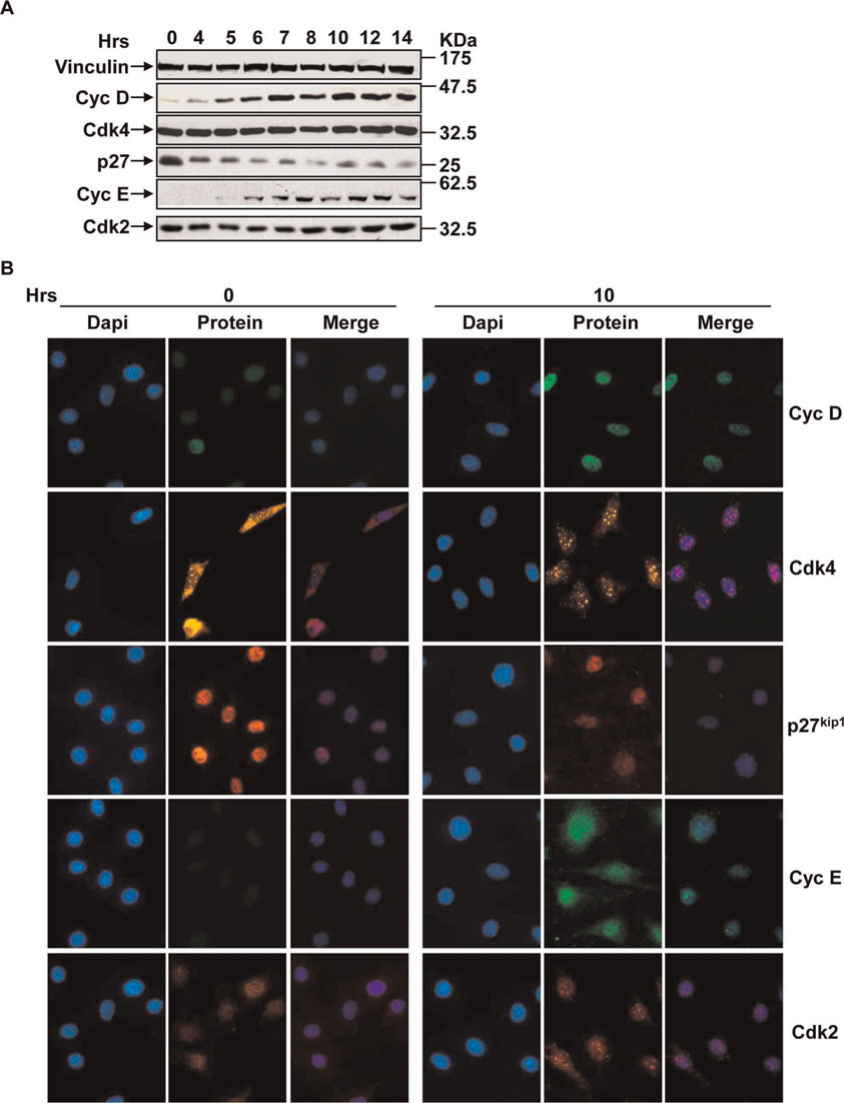
**Expression and localization of cell cycle proteins in G_1 _to S transition**. (**A**) Time-courses of the expression of proteins involved in the control of G_1 _to S transition. NIH3T3 cells, made quiescent by serum starvation, were stimulated with 10% serum and collected at appropriate time points and total cellular extracts were subjected to SDS-PAGE followed by Western blotting with appropriate antibodies. The Western blot is representative of at least three independent experiments. (**B**) Localization of proteins involved in the control of G_1 _to S transition at time 0 and 10 hours. NIH3T3 cells, after synchronization by serum starvation, were labeled with indicate antibodies (protein) and analyzed by fluorescence microscope. Nuclei were visualized by DAPI staining. The merged images are the result of a merge between the two single images acquired. At least 200 cells were scored for each sample and the images are representative of three independent experiments.

Then, the content of several cell cycle proteins was estimated (over equal amounts of cellular proteins) by Western blot analysis (Figure 3A). It is clear that quiescent cells (at time 0) are characterized by very low levels of cyclin D and cyclin E, by sizable levels of Cdk4, Cdk2 and Cki p27^Kip1^. At 4–6 hrs the level of cyclin D substantially increases, while that of p27^Kip1 ^starts to decrease. At 6–8 hrs cyclin E starts to be detectable, while p27^Kip1 ^disappears almost completely. The localization analysis (Figure 3B) indicates that in quiescent cells the great majority of p27^Kip1 ^is localized into the nucleus, while Cdk4 and Cdk2 are localized both in the nucleus and in the cytoplasm.

### Parameter estimation, computational analysis and simulated dynamics of key players during the G_1 _to S transition

**Figure 4:**
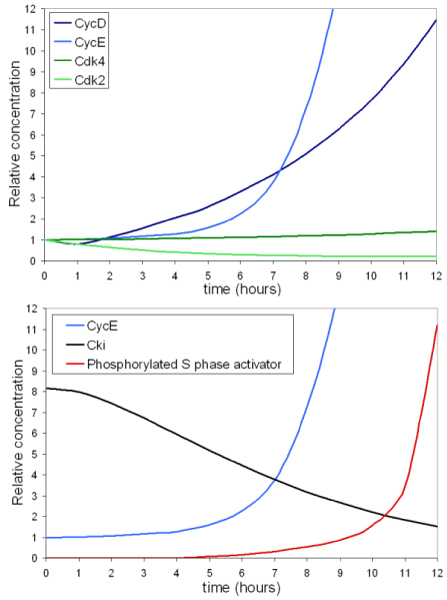
**Simulated time courses for the total concentrations of G_1 _to S transition key players**. Global dynamics describing the concentrations in time are reported for major cell cycle players. The phosphorylated S phase activator (the final output of the system whose level is proportional to probability of starting S phase) is also shown.

The model was constrained to fit the observed experimental behavior of the G_1 _to S transition (Figure 2) and of relevant players considered in the network (Figure 3). Rate constants and values at the beginning of the simulations (reported in Additional file 2 and Additional file 3, respectively) were derived considering both the experimental values described above and literature data as described in the Appendix. Cells resume growth with their characteristic exponential rate (duplication time = 24 hours) as soon as growth factors are added. Results of a 12 hours simulation of the G_1 _to S transition are reported in Figure 4. Levels of both Cdk4 (dark green) and Cdk2 (light green) show little change over the time frame considered in our simulations (Figure 4A), consistently with experimental data (Figure 3). The rise in cyclin D1 (dark blue) is a quite early event and is followed a few hours later by a rise of cyclin E (light blue). Has to be noted that since we are simulating the G_1 _to S transition and not a full cell cycle, time courses of some variables become meaningless after entrance into S phase. Simulation results for Cki (Figure 4B, black line) show progressive time-dependent degradation. Similarly, the level of p27^Kip1 ^appears to diminish smoothly within the first part of the serum-stimulation experiment, then it disappears almost completely as shown by both Western blot and immunofluorescence (Figure 3A and 3B, respectively). The final output of the G_1 _to S simulation, i.e. the phosphorylated S phase activator starts to be present at 8–9 hours (Figure 4, red line), consistent with the timing experimentally determined. In summary, considering both the final output of the system (i.e. propensity to enter into S phase) and the dynamics of selected key components, the dynamics of the system are in agreement with experimental data shown in Figures 2 and 3 and with literature data discussed in more detail in the Appendix.

### Simulated localization of cycle proteins and complexes

**Figure 5:**
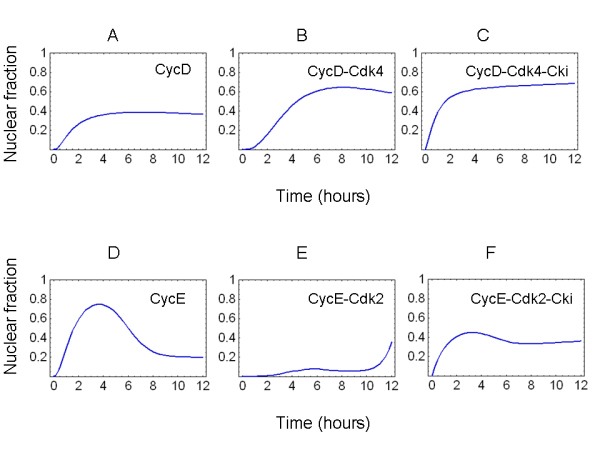
**Nuclear localization of cyclins and their binary and ternary complexes**. The nucleo/cytoplasmic ratio for cyclins and the relative binary and ternary complexes is shown. Results are shown for cyclin D and cyclin E (panels **A **and **D**, respectively), for cyclin D/Cdk4 and cyclin E/Cdk2 active complexes (panels **B **and **E**, respectively) and for the ternary complexes, cyclin D/Cdk4/Cki and cyclin E/Cdk2/Cki (panels **C **and **F**, respectively).

Immunofluorescent localization data shown in Figure 3B indicate that cyclins, Cdks and Cki can be detected in the nucleus. Indeed, in order to be able to phosphorylate their targets, Cdk-containing complexes must efficiently reach the nucleus, i.e. a sizable fraction of each complex must be present within the nucleus. Figure 5 reports the simulated fraction of nuclear cyclin D (panel A) and cyclin D-containing binary and ternary complexes (panels **B **and **C**, respectively) in the upper row, while the bottom row displays the simulated fraction of nuclear cyclin E (panel **D**) and cyclin E-containing binary and ternary complexes (panels **E **and **F**, respectively). Both cyclins and their complexes are efficiently transported to the nucleus, despite the fact that at time 0 cyclins are found only in the cytoplasm where they are synthesized, indicating that the transport kinetics implemented in the model are able to efficiently drive the system.

### The carrier-function of Cki: gene dosage effects

**Figure 6:**
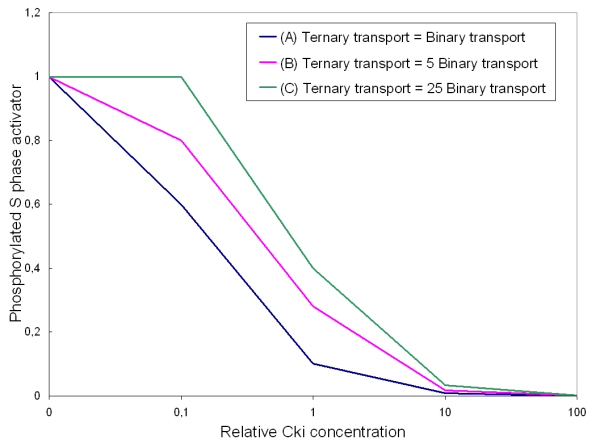
**S phase entrance rate *vs *Cki relative concentration depending on the translocation of binary and ternary cyclin/Cdk complexes**. The level of phosphorylated S phase activator at the end of the simulation (taken as a measure of the G_1 _to S transition) is shown as a function of relative Cki concentration at the beginning of the simulation. Three cases are presented: rate constants for the cytoplasm-to-nucleus translocation of binary and ternary cyclin/Cdk complexes are equal (blue line); the rate constants for the ternary complexes is 5-fold higher compared to the binary complexes translocation constants (pink line, corresponding to our standard simulation parameter set); the rate constants for the ternary complexes is 25-fold higher compared to the binary complexes translocation constants (green line).

It has been reported that p27^Kip1 ^over-expression correlates with cell cycle arrest [35,36] and, conversely, that its degradation is a key pre-requisite for entry into S phase [37]. At the same time, a role for Ckis in promoting nuclear transport and/or assembly of cyclin/Cdk complexes has also been shown [38,39]. Such a dual role has been incorporated in our model since: (i) only nuclear cyclin/Cdk complexes – but not cyclin/Cdk/Cki complexes – are able to phosphorylate relevant substrates and (ii) cyclin/Cdk/Cki ternary complexes enter the nucleus 5-fold faster than corresponding cyclin/Cdk binary complexes (see Additional file 2). As noted above, in our model the entrance into S phase is accounted for as phosphorylation of the S phase activator, assuming the S phase entrance to be proportional to the level reached by the phosphorylated activator at the end of the simulation, i.e. 12 hours after "serum stimulation". Accordingly, Figure 6 reports the effects of changing Cki concentration on S phase entrance, when the rate constants for nuclear transport of ternary cyclin/Cdk/Cki complexes are the same (dark blue line), 5-fold higher (the condition considered as standard in our model, pink line) and 25-fold higher (green line) than those of the binary cyclin/Cdk complexes. Cki concentration is shown on a log scale, taking as 1 the Cki concentration used in our reference set. If the Cki has no transport-promoting effect, i.e. if cyclin/Cdk/Cki ternary complexes enter the nucleus as fast as the corresponding cyclin/Cdk binary complexes, increasing Cki concentration has a purely negative effect on the entrance into S phase (dark blue line). The inhibitory effect is partially overcome when cyclin/Cdk/Cki ternary complexes enter the nucleus 5-fold (pink line) or 25-fold (green line) faster than corresponding cyclin/Cdk binary complexes. The S phase promoting effect is more evident at lower Cki concentration and is completely lost at a relative concentration of 10 or higher when S phase entrance is completely shut-off, regardless of whether there is any advantage for the transport of the ternary complexes over the binary complexes or not.

### Comparison of simulated and experimental overcoming of the restriction point

The R point was experimentally determined as follows. Quiescent cells were exposed to serum for variable periods of time and then transferred to serum-free medium. The fraction of BrdU-positive cells was measured at the end of the incubation period (14 hours) and is plotted in Figure 2A in correspondence of the time spent in the presence of serum. While cells exposed for only 2 hours were not able to enter S phase, almost 40% of cells stimulated with serum for 6 hours were BrdU-positive, i.e. entered S phase when assayed at 14 hours, after 8 hours of serum starvation.

The corresponding computational experiment involved running the simulation with parameters and starting conditions reported in Additional files 2 and 3 (**"ON" **condition) for different times, stopping the run, and re-starting it with cyclin D synthesis shut-off, cyclin D degradation increased and Cki synthesis turned-on ("**OFF**" condition), to simulate growth factor removal. Starting conditions of the second part of the simulation for all variables corresponded to those reached when "**ON**" condition simulation was first stopped. Ability to enter S phase was estimated as described in the previous paragraph. Values of the phosphorylated S phase activator at the end of each 12 hours run (total "**ON**" + "**OFF**" simulation time) are plotted as a function of the simulation time in the "**ON**" condition.

**Figure 7:**
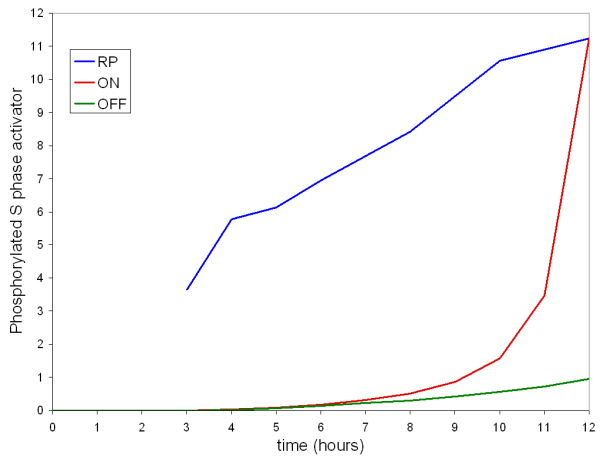
**Simulated Restriction point dynamics**. Simulated accumulation of the phosphorylated S phase activator (taken as a measure of the G_1 _to S transition) upon growth factor stimulation ("**ON" **condition for the whole length of the simulation, red line), for a simulation run continuously in the "**OFF**" condition (green line) and summary of simulations run for R point determination (blue line). These simulations have been run for an increasing number of hours in the "**ON**" condition and for the time remaining to 12 hours in the "**OFF**" condition. Values of the phosphorylated S phase activator at the end of each 12 hours (total "**ON**" + "**OFF**" time) run are plotted as a function of the simulation time in the "**ON**" condition.

Figure 7 compares accumulation of the phosphorylated S phase activator (i) for a regular simulation ("**ON" **condition for the whole length of the simulation, red line), (ii) for a simulation run continuously in the "**OFF**" condition (green line) and (iii) for simulations run for R point determination (blue line). Note that output of the system is quite sharp, but is not describing the S phase status of each individual cell (a yes/no function), but rather the probability to enter into S phase that increases as the phosphorylated activator builds-up. The half-maximal value of the line describing the R point estimation (blue line) is reached after 4–5 hours. This value agrees with the corresponding experimental values well as with the value determined by deconvolution (Figure 2A) and is in the same range as the values reported by other authors for NIH3T3 cells [30].

## Conclusion

The cell cycle is a complex process that allows eukaryotic cells to replicate DNA and partition it into two daughter cells. Its regulation is exceedingly complex and must take into account – and integrate – intracellular and extracellular signals. Multi-cellular organisms must also coordinate cell cycle of their component cells in order to keep harmonic and functional arrangement of tissues and organs. Such a coordination mostly take place within the G_0_/G_1 _phase at a point called the restriction (R) point [40]. Alteration in the R point and ensuing inability to coordinate entry into the cell cycle with mitogenic and nutritional signals leads to unregulated proliferation and ultimately to cancer [41].

Such a central physio-pathological role of cell cycle has stimulated a wealth of computational studies aimed to capture the logic of its functioning through mathematical analysis of the specific molecular mechanism involved in the process. Different mathematical models, specifically focused on the G_1 _to S transition in the mammalian cells, have been reported [42-47] and each one uses a specific approach and/or focuses on specific components to simulate the cell cycle dynamics. These include models published by: Kohn [47] whose core module is the E2F-pRb complex; Aguda and co-workers [43] whose R point model involves both D-type and E-type cyclins, and their cognate kinases Cdk4 and Cdk2, the inhibitor p27^Kip1^, and the mitogenic signals in the R-point transition; Qu and collaborators [44] in 2003, whose model considers multiple phosphorylation sites for the components involved in the regulation of the G_1 _to S transition, which is a crucial point in the cell cycle process and strengthens the involvement of E2F1/pRB and Cdc25A in R point execution; Swat and co-workers [45] whose model considers only few components in the G_1_/S transition, but they aim to identify the small feedback loops in the regulation process of the R-point transition in terms of bifurcation analysis; Haberichter and collaborators [46] whose model is based on the presence of an unknown "modifier" that activates Cdk2 in response to metabolic signals; similar to the proposal of Tyson and Novak [20] they assign an important role in the G_1 _to S transition to A-type cyclins, that are reported in literature to be required more for the execution – rather than for the onset – of S phase [48-52].

A generic model for the restriction point control of the mammalian cell cycle was presented by Novak and Tyson in 2006 [24]. Neither their model, nor subsequent extensions [20] nor the other cell cycle models presented so far explicitly consider nucleus/cytoplasm localization.

The major novelty of the mathematical model of entry of quiescent mammalian fibroblasts into S phase upon stimulation by growth factors that we present here is the explicit account of the nucleo/cytoplasmic localization of cell cycle players that shuttle between the two compartments as well as of cell growth. Consistently with the notion that the core cell cycle machinery appears to be conserved in all eukaryotes, from yeast to human [19], our mathematic model is based on the network of the G_1 _to S transition in budding yeast [26] that explicitly considers nucleus/cytoplasm localization. Besides, the choice of parameters of our model has been constrained by experimental data obtained on murine fibroblasts synchronized by serum starvation and stimulated by serum to enter S phase, while the several models previously described [43-45,47] were based only on theoretical considerations or experimental data taken from literature.

It is worthwhile to underline similarities and differences among the G_1 _to S network of budding yeast [26] and mammals (this paper). The yeast model incorporated two Ckis, while only a single Cki is considered in the present mammalian model. As outlined above, the single Cki more closely resembles p27^Kip1^, whose role in cell proliferation and cancer is well established, purposefully neglecting p21^Cip1 ^and inhibitors of the Ink family [53]. Both models incorporate a dual (inhibitory and stimulatory through promotion of cyclin/Cdk translocation) role for a Cki, namely Sic1 in budding yeast and p27^Kip1 ^in mammals. Functional equivalence between yeast and mammalian cyclin complexes in our models is as follows: Cln3/Cdk1 to cyclin D/Cdk4,6; Clb5,6/Cdk1 to cyclin E/Cdk2, slightly different from that proposed by other authors [20]. In the mammalian model, no equivalent of the yeast Cln1,2/Cdk1 complex is present, given the fact that the Cln/Cdk complexes play a major role in promoting budding, that is a yeast-specific process. Functional equivalence between yeast and mammalian cyclin/Cdk complexes have been reported by other authors. In the yeast model [26] and in the present one, a relevant role is played by cyclin/Cdk phosphorylation of functionally equivalent inhibitors (Whi5 in yeast and Rb in mammalian cells) that originates a free transcription factor (SBF/MBF in yeast, E2F in mammalian cells) that drives transcription of genes required to enter S phase. While the molecular logic is the same, some mechanistic details differ, since a single kinase (Cln3/Cdk1) phosphorylates Whi5 in yeast, while cyclin D/Cdk4,6 and cyclin E/Cdk2 sequentially phosphorylate the Rb inhibitor in mammals.

Simulations of the model allows to recapitulate events happening from growth factor stimulation (occurring at time 0 of the simulation) and shows successive building-up of cyclin D and cyclin E with a timing consistent with the experimental ones. Active complexes are found in the nucleus at appropriate times and building-up of the phosphorylated S phase activator is also consistent with experimental data. Removal of the growth factor (that is simulated by turning off cyclin D synthesis and increasing cyclin D degradation and Cki synthesis) allows to construct a restriction point curve that is similar to the one experimentally determined. It should be noted here that experimental curves are obtained on a cell population, i.e. they represent the fraction of BrdU-positive cells, while simulated curves not only refer to a single cell, but they do not show the (discontinuous) onset of S phase, but phosphorylation of a S phase activator whose continuous accumulation will allow trigger (discontinuous) entry into S phase.

**Figure 8:**
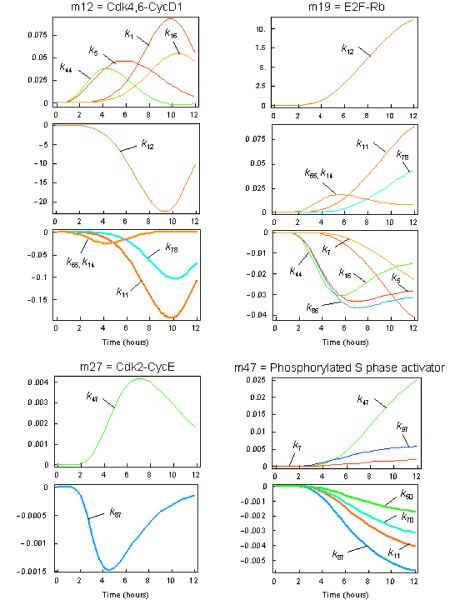
**Sensitivity analysis of the G_1 _to S network**. To test the impact of the parameter values on the dynamic behavior of the system, sensitivity analysis was performed by calculating so-called time-dependent response coefficients *R *= (∂*c*_i _(*t*)/*c*_i _(*t*))/(∂*p/p*) that allow to trace the time-dependent effect of a parameter change on a concentration during the whole simulation period. See Additional file 1 for a list of parameters.

Cki inhibitors have been proposed to define thresholds for cyclin/Cdk activity by setting levels that cyclin-Cdk complexes must exceed to become active [54]. According to this notion, cell cycle progression or arrest would depend on relative concentration of inhibitors and cyclins: a decrease in cyclin/Cdk components or an increase in inhibitor levels would prevent the accumulation of inhibitor-free cyclin/Cdk complexes, thus inhibiting cell cycle progression. The sensitivity analysis reported in Figures 6 and 8 is consistent with this notion, but also strongly underlines the biological relevance of the shuttling role of the Cki indicating that our model is going to test on a quantitative basis this assumption. Figure 6 indicates that the effect brought about by Cki overexpression is strictly dependent on whether or not the Cki is promoting nuclear translocation of cyclin/Cdk containing complexes. Figure 8 shows that Cki-related constants (k_7_, k_11_, k_12 _and k_78_), that positively promote formation of the cyclin D/Cdk4,6 complex, affect negatively E2F-Rb (i.e. promote its dissociation that is a prerequisite for S phase onset). Constants k_7 _e k_11 _also affect the time course of the production of the phosphorylated S phase activator, i.e. the final output of our system. Together, these results show that alterations of the Cki dynamics (initial level, degradation, rate of nuclear transport, ability to promote translocation of cyclin-containing complexes) deeply affects the ability of quiescent cells to respond to growth factors.

Further development of the model may include a more sophisticated mathematical analysis of Rb phosphorylation. Our current model only implements two sequential phosphorylations, while patterns of Rb phosphorylation are quite complex (see Appendix for details and references). Thanks to their ability to act as signaling switches and counting mechanism [55,56], implementation of multisites protein phosphorylation of Rb may contribute to render the G_1 _to S transition steeper and closely resembling an "on/off" switch [57]. The same effect would be obtained by direct implementation of the molecular mechanism linking phosphorylation of the S phase activator to S phase triggering that involves its association with the Origin Recognition Complex (ORC) at replication origins and subsequent recruitment of other initiation factors, like the MCM proteins [58-60].

## Methods

### Deterministic model for concentration changes

The dynamics are described by ordinary differential equations (ODEs) using mass action kinetics. Cell growth is characterized as exponential increase in volume, and all concentration changes are dependent on the volume changes of the respective compartment. We explicitly consider two compartments, the nucleus and the cytoplasm. See Barberis et al. [26] for a detail description.

### Estimation of parameter values and rate constants

The model comprises 98 rate constants, shown in Additional file 2. Rate constants for cell growth (k_114_) as well as the ratio between the nuclear and the cytoplasmic volumes, were experimentally measured in the NIH3T3 murine fibroblasts. Care was taken to keep biologically similar rate constants within the same range, unless available data suggested otherwise. So for instance, rate constants for E2F-dependent productions (Cdc25A, cyclin E, E2F itself) were very similar (2; 2 and 1 (pM^-1^*h^-1^), respectively). The same criteria were applied to rate constants for production, degradation, association and dissociation (note that to reduce the number of parameters several association or dissociation reaction were considered irreversible). For the cytoplasmic/nuclear transport, the rate of nuclear transport of ternary cyclin/Cdk/Cki complex was set to be 5-fold that of the corresponding binary complexes (see text for details). Choice of parameters was also constrained by fitting to the experimental data.

### Sensitivity analysis

Sensitivity analysis was performed to test the influence of the parameter choice on the systems dynamics. To this end, we calculated the time-dependent response coefficients [61] defined as

R = (∂c_i _(t)/c_i _(t))/(∂p/p). These coefficients indicate the direction and amount of change of the time course for the concentration c(t) upon an infinitesimal change of the parameter (or initial concentration) p. Loosely spoken, one can also interpret this as the percentage change of the concentration over time upon a 1% change of the parameter. During model development, the response coefficients were used to indicate appropriate parameter changes, since there are not enough data available to estimate the parameters by a global approach.

### Cell culture

Mouse embryonic fibroblast NIH3T3 cells (CRL-1658; American Type Culture Collection)[59], were routinely grown and maintained in culture as previously described [62].

### Cell synchronization

Cell synchronization was performed as previously described [62].

In order to identify the restriction point in our cellular model of murine fibroblasts NIH3T3, the cells were synchronized as previously described, then stimulated with serum for variable times at which the cells were re-starved and cultured until 15 hours post-release in the presence of BrdU. The percentage of the cells able to enter in S phase, following the re-starvation, was scored by flow cytometry as described below. As control of entry in S phase, we used the cells released in a complete medium plus BrdU and analyzed at 15 hours post-stimulation.

### Flow cytometric analysis

The distribution of cells at specific cell cycle phases was evaluated by flow cytometry as previously [62].

### Immunofluorescence microscopy

Immunofluorescence microscopy analysis of protein localization was performed using methods previously described [63].

### Immunoblot analysis

Western blot analysis for identification of protein expression was performed as previously described [62,64].

## Appendix

### Data mining to determine the wiring diagram of the G_0_/G_1 _to S transition

The interaction pattern of the model components involved in the G_1 _to S transition process was performed through a literature and web-based data-mining. This process required an extensive literature searching using electronic resources, such as PubMed from NCBI and, furthermore, a wide web-based search was necessary in order to identify the main protein-protein interaction involved in the processes. This step implied the browsing of many different bioinformatics databases, such as protein-protein interactions resources (BIND [65], Mint [66], IntAct [67]), cell cycle specific database (Cell Cycle Database [68]) and pathway resources such as the Kegg Pathway and Reactome data-bases [69,70]. When the model components and their interaction have been identified, a wiring diagram of the G_1 _to S transition has been drawn using CellDesigner [27-29], a structured diagram editor for drawing gene-regulatory and biochemical networks that are stored using the Systems Biology Markup Language (SBML).

### Function and regulation of cyclin D, Cdk4/6 and Ckis

Three cyclin D isoforms (D1, D2, and D3), with similar functions during the G_1 _phase of the cell cycle have been described [71]. Cyclin D1 is a key sensor and integrator of extracellular signals from early to mid-G_1 _phase [72] that acts primarily through its ability to turn on specific Cdks required in the G_1 _phase [73,74], cyclin D being mainly found in complex with Cdk4,6 proteins [71,74,75]. Growth factors and hormones, in a cell type specific manner, regulate the expression of cyclin D1 [71,74-76]. Cyclin Ds levels are low in quiescent cells and rise progressively during early G_1 _phase in response to stimulation by growth factors [77]. To simplify the model implementation we assume the presence of a generic "modifier" able to regulate cyclin D synthesis in response to mitogenic stimulation. Cdk4,6 proteins, whose level in quiescent cells is sizeable, are present in non-limiting amount following growth factors stimulation [72,74].

The cyclin D/Cdk4,6 complexes play key roles in regulating G_0 _to S phase transition through at least two different mechanisms: phosphorylation of specific substrates required for the G_1 _to S transition [77] and sequestration of p21^Cip1^/p27^Kip1 ^inhibitors from cyclin E-Cdk2 complexes to avoid premature S phase activation [77]. Kinase activity of cyclin D/Cdk4,6 complexes, increases from mid-G_1 _and reaching a maximum in close proximity to the G_1 _to S boundary [74,78], the major substrate being Rb proteins in complex with the transcription factor E2F [79,80].

Interaction of Ckis with cyclin D/Cdks in the execution of the G_0_/G_1 _to S progression with the Cdk inhibitors (Cki), p21^Cip1 ^and p27^Kip1 ^[54] also promotes stabilization and activity of cyclin D/Cdks complexes themselves. The levels of the two Ckis are quite different in quiescent cells: expression of p27^Kip1 ^is high in quiescent cells, since the synthesis and the stability of p27^Kip1 ^is inhibited by mitogenic signals [81], while the expression of p21^Cip1 ^is lower than p27^Kip1 ^in quiescent cells and frequently increases during G_1 _phase progression in response to mitogenic signals [82,83]. Hence both inhibitors, albeit at low level, are present along the G_0_/G_1 _to S transition. Data obtained in MEF cells lacking p21^Cip1 ^and/or p27^Kip1 ^genes, have clearly shown that both proteins promote processes that positively regulate cell cycle progression, such as cyclin D assembly with Cdk4, stability and nuclear localization [38]. Finally, it has been shown that cyclin D/Cdk activity is required for re-entry of resting cells into the cell cycle and cannot be fully compensated by cyclin E/Cdk activity in p21/p27-null MEFs.

### Regulation of E2F transcription factor activity

The major role of the G_1 _cyclin D/Cdk4,6 and cyclin E/Cdk2 complexes in controlling G_1 _to S phase progression is the inactivation of Rb that loosens its inhibitory interaction with transcription factor E2F. Several genes encoding proteins that are essential for cell proliferation, including genes encoding as the cyclins E and A, proteins essential for DNA replication, such as DNA polymerase, thymidine kinase, dihydrofolate reductase, and histone H2A, as well as E2F itself, are controlled at least in part by E2F-responsive promoters. In quiescent cells (G_0 _phase) Rb is unphosphorylated [84,85] while serum promotes its phosphorylation. In the wiring diagram that we have constructed and in keeping with literature data, Rb phosphorylation occurs in two sequential steps: the first step consists in the phosphorylation of Rb from the active cyclin D1/Cdk4,6/Cki complex [80], which leads to a so-called hypo-phosphorylated Rb, i.e. a Rb form that has been shown *in vivo *to be phosphorylated on 13 of 16 potential Cdk phosphorylation sites, suggesting that it may consist of multiple phosphoisoforms [86]. Hypophosphorylation of Rb in early G_1 _stimulates the release of histone deacetylase 1 and the recruitment of transcription factors of the SWI/SNF family to the Rb-containing chromatin remodeling complexes, thus allowing the expression of cyclin E. The second step takes place in late G_1 _and during the S phase. It is catalyzed by cyclin E/Cdk2 complexes (and later by cyclin A/Cdk2 complexes, not included in our model) [87-89]. It originates the so-called hyper-phosphorylated Rb that looses even more affinity for – and therefore fails to inhibit the transcriptional activation activity of – E2F. When freed from Rb, E2F activates transcription of genes encoding cyclin E and Cdk2, thereby promoting synthesis of their encoded proteins. Similarly, E2F promotes the synthesis of Cdc25A and Cdc6, which have the role of activating the cyclin E/Cdk2 complex and promoting the onset of DNA replication respectively [90-93] as well as of E2F itself [49].

### Cyclin/Cdk complexes localization during G_0_/G_1 _to S transition

An important determinant for the G_1 _to S transition, is the localization of the two cyclin/Cdks complexes. Because neither cyclin D1 nor Cdk4 has a recognizable nuclear localization sequence, the mechanisms governing cyclin D1 nuclear import remain undefined. Some authors suggested that nuclear export of cyclin D1 is a major determinant of cyclin D1/Cdk4 localization [94]. Indeed phosphorylation of cyclin D1 at a single threonine residue, Thr-286, by GSK-3 facilitates the binding of cyclin D1 with the nuclear exportin, CRM1, and thereby promotes cyclin D1 nuclear export [94]. This process can be inhibited by p21^Cip1^, that interacting with cyclin D1, abolishes cyclin D1-CRM1 association inducing cyclin D1 nuclear accumulation [94]. Unlike cyclin D1, both p21^Cip1 ^and p27^Kip1 ^contain canonical nuclear localization signal motifs [36,95] and can promote the nuclear accumulation of cyclin D1/Cdk4 complexes in transient transfection experiments [38,39,96]. However, although p21^Cip1 ^can facilitate the nuclear accumulation of cyclin D1, the loss of both p21^Cip1 ^and p27^Kip1 ^does not abolish cyclin D1 nuclear import [38], thus, neither p21^Cip1 ^nor p27^Kip1 ^are strictly required for cyclin D1 nuclear import. The finding that both p21^Cip1 ^and p27^Kip1 ^are components of active cyclin/Cdk complexes [39,97,98], that p21^Cip1 ^can promote the assembly of cyclin D/Cdk4 complexes *in vitro *[39] and that p21^Cip1 ^and p27^Kip1 ^can be involved as nuclear import factors for the cyclin D1/Cdk4 complex, has rendered more intriguing their role in cell cycle.

The first event that our model indicates to occur in the cytoplasm is the formation of the binary complex between cyclin D1 and Cdk4,6 proteins, immediately followed by the formation of a ternary complex with the binding of Ckis to the binary complex. The ternary complex goes into the nucleus driven by the Cki Nuclear Localization Sequence (NLS). Here, the complex is activated by the Cdk activating kinase Cak, which phosphorylates Cdk4,6, and the active ternary complex is now able to phosphorylate Rb in complex with E2F, promoting partial release of E2F leading to transcriptional activation of its cognate genes. A parallel event of ternary complex formation into the nucleus is also considered: that is the cyclin D1/Cdk4/6 complex enters into the nucleus without Cki and binds to the inhibitors into the nucleus.

The second important event occurring in the cytoplasm, several hours later the formation of the cyclin D/Cdk4,6 complex, is the formation of the cyclin E/Cdk2 binary complex. The binary cyclin E/Cdk2 complex is able to translocate into the nucleus thanks to the NLS of cyclin E [99]. However, as soon as this complex is formed, Cki binding occurs forming a ternary complex. After nuclear translocation, the ternary complex dissociates releasing the Cki, and the cyclin E/Cdk2 binary complex is sequentially phosphorylated by two kinases: Wee1 (inhibitory) and Cak activating). Activation of the binary complex is achieved through the action of the phosphatase Cdc25A that removes the Wee1 inhibitory phosphorylation. The active cyclin E/Cdk2 binary complex promotes hyperphosphorylation of Rb, which is in complex with E2F, in the way that E2F dissociates from Rb promoting the DNA synthesis process.

### Onset of DNA replication

DNA replication is a regulated process strictly coupled to the progression of the cell cycle, the initiation of DNA replication occurring at discrete chromosomal replication origins. Many proteins are involved in the initiation of DNA replication. In our model, we consider that a nuclear S phase activator is phosphorylated in a cell cycle-dependent manner by the active cyclin E/Cdk2 binary complex. Phosphorylation of the S phase activator is the final event included in our network and initiation of DNA replication taken to be proportional to its build-up.

## Competing interests

The authors declare that they have no competing interests.

## Authors' contributions

RA and MB Data mining, mathematical implementation of the network, running and analysis of simulations, sensitivity analysis (MB); writing of the paper; DG and FC Data mining, planning and execution of experiments on mammalian cells; writing of the paper (FC); LM Supervision of mathematical modeling and simulation; MV Supervision of network identification; quality control, debugging and evaluation of the biological significance of the simulations; writing of the final version of the paper; EK Supervision of mathematical modeling and simulation; LA Planning of the experiments; supervision of network identification; evaluation of the biological significance of the simulations; writing of the final version of the paper.

## Supplementary Material

Additional file 1 Set of kinetic equations (a) and Ordinary Differential Equations (ODE) (b) describing the mathematical model of the G1/S transition. Click here for file

Additional file 2 Rate constants in the wild type condition. In bold are the parameters altered in the *off state *(in parentheses are reported the values for the *off state*). Click here for file

Additional file 3 Initial concentration values in both wild type and *off state*. Click here for file
