# Supplementary material for: Towards a systems biology approach to mammalian cell cycle: modeling the entrance into S phase of quiescent fibroblasts after serum stimulation
Source: BMC Bioinformatics. 2009 Oct 15;10(Suppl 12):S16. doi: 10.1186/1471-2105-10-S12-S16 (PMC2762065; doi:10.1186/1471-2105-10-S12-S16)
Supplement: Additional file 1 — Set of kinetic equations (a) and Ordinary Differential Equations (ODE) (b) describing the mathematical model of the G1/S transition. [file 1471-2105-10-S12-S16-S1.doc]

Additional file 1. Set of kinetic equations (a) and Ordinary Differential Equations (ODE) (b) describing the mathematical model of the G1/S transition.

**(a) Kinetic equations**

| **Kinetic equation** | **Description** |
| --- | --- |
|  | **Protein synthesis** |
| v[1][t] = k_1_*m[51][t] | Cdk4/6 synthesis mediated by a generic serum-dependent modifier |
| v[3][t] = k_3_*m[51][t] | Cyclin D synthesis mediated by a generic serum-dependent modifier |
| v[6][t] = k_6_ | Cki synthesis |
| v[13][t] = k_13_ | CAK synthesis |
| v[18][t] = k_18_*m[51][t] | GSK3beta activation mediated by a generic serum-dependent modifier |
| v[24][t] = k_24_ | Rb synthesis |
| v[26][t] = k_26_ | E2F synthesis |
| v[30][t] = k_30_ | PP1 synthesis |
| v[35][t] = k_35_ | PP2A synthesis |
| v[46][t] = k_46_ | S phase activator synthesis |
| v[47][t] = k_47_ | Cyclin E synthesis |
| v[48][t] = k_48_ | Cdk2 synthesis |
| v[61][t] = k_61_ | Wee1 synthesis |
| v[64][t] = k_64_ | CAK synthesis |
| v[67][t] = k_67_ | Cdc25A synthesis |
| v[83][t] = k_83_*m[51][t] | Generic serum-dependent modifier synthesis |
|  |  |
|  | **Protein degradation** |
| v[2][t] = k_2_*m[1][t] | Cdk4/6 degradation |
| v[4][t] = k_4_*m[2][t] | Cyclin D degradation |
| v[7][t] = k_7_*m[4][t] | Cki degradation |
| v[14][t] = k_14_*(m[9][t]+m[44][t]) | CAK degradation |
| v[17][t] = k_17_*m[50][t] | Cki-P nuclear degradation |
| v[19][t] = k_19_*m[13][t] | GSK3beta degradation |
| v[22][t] = k_22_*m[15][t] | Cdk4/6-P degradation |
| v[23][t] = k_23_*m[16][t] | Cyclin D-P degradation |
| v[25][t] = k_25_*m[17][t] | Rb degradation |
| v[27][t] = k_27_*m[18][t] | E2F degradation |
| v[31][t] = k_31_*m[20][t] | PP1 degradation |
| v[34][t] = k_34_*m[22][t] | Rb-P degradation |
| v[36][t] = k_36_*m[23][t] | PP2A degradation |
| v[39][t] = k_39_*m[25][t] | Rb-P-P degradation |
| v[42][t] = k_42_*m[26][t] | E2F degradation |
| v[50][t] = k_50_*m[31][t] | S phase activator degradation |
| v[53][t] = k_53_*m[34][t] | Cyclin E degradation |
| v[54][t] = k_54_*m[35][t] | Cdk2 degradation |
| v[60][t] = k_60_*m[41][t] | Cki-P degradation |
| v[62][t] = k_62_*m[43][t] | Wee1 degradation |
| v[65][t] = k_65_*(m[9][t]+m[44][t]) | CAK degradation |
| v[68][t] = k_68_*m[30][t] | Cdc25A degradation |
| v[70][t] = k_70_*m[46][t] | Cdc25A-P degradation |
| v[74][t] = k_74_*m[48][t] | Cdk2-P degradation |
| v[75][t] = k_75_*m[49][t] | Cyclin E degradation |
| v[84][t] = k_84_*m[51][t] | Generic serum-dependent modifier degradation |
|  |  |
|  | **Complex formation** |
| v[5][t] = k_5_*m[1][t]*m[2][t] | Cyclin D-Cdk4/6 cytoplasmic complex |
| v[8][t] = k_8_*m[3][t]*m[4][t] | Cyclin D-Cdk4/6-Cki cytoplasmic complex |
| v[12][t] = k_12_*m[12][t]*m[8][t] | Cyclin D-Cdk4/6-P-Cki nuclear complex |
| v[28][t] = k_28_*m[17][t]*m[18][t] | Rb-E2F complex |
| v[55][t] = k_55_*m[34][t]*m[35][t] | Cyclin E-Cdk2 cytoplasmic complex |
| v[76][t] = k_76_*m[4][t]*m[36][t] | Cyclin E-Cdk2-Cki cytoplasmic complex |
| v[87][t] = k_87_*m[8][t]*m[27][t] | Cyclin E-Cdk2-P-Cki nuclear complex |
|  |  |
|  | **Complex dissociation** |
| v[16][t] = k_16_*m[10][t] | Cyclin D-Cdk4/6-Cki cytoplasmic complex |
| v[21][t] = k_21_*m[14][t] | Cyclin D-P-Cdk4/6-P cytoplasmic complex |
| v[33][t] = k_33_*m[21][t] | E2F-Rb-P complex: partial dissociation of Rb-P from E2F |
| v[38][t] = k_38_*m[24][t] | E2F-Rb-P-P complex: complete dissociation of Rb-P-P from E2F |
| v[44][t] = k_44_*m[7][t] | Cyclin D-Cdk4/6-Cki nuclear complex |
| v[58][t] = k_58_*m[39][t] | Cyclin E-Cdk2-Cki nuclear complex |
| v[73][t] = k_73_*m[27][t] | Cyclin E-Cdk2 complex |
|  |  |
|  | **Cytoplasm-to-nucleus translocation** |
| v[9][t] = k_9_*m[3][t] | Cyclin D-Cdk4 complex |
| v[10][t] = k_10_*m[5][t] | Cyclin D-Cdk4-Cki complex |
| v[11][t] = k_11_*m[4][t] | Cki |
| v[56][t] = k_56_*m[36][t] | Cyclin E-Cdk2 complex |
| v[77][t] = k_77_*m[37][t] | Cyclin E-Cdk2-Cki complex |
|  |  |
|  | **Nucleus-to-cytoplasm translocation** |
| v[49][t] = k_49_*m[28][t] | Cyclin E mRNA |
| v[78][t] = k_78_*m[8][t] | Cki |
|  |  |
|  | **Phosphorylation reactions** |
| v[15][t] = k_15_*k_100_*m[6][t]*(m[9][t]+m[44][t]) | Cdk4/6 phosphorylation by CAK |
| v[20][t] = k_20_*k_101_*m[12][t]*m[13][t] | Cyclin D phosphorylation by GSK3beta |
| v[29][t] = k_29_*k_102_*m[10][t]*m[19][t] | Rb partial phosphorylation by Cyclin D-Cdk4/6-P-Cki |
| v[40][t] = k_40_*k_103_*m[27][t]*m[21][t] | Rb complete phoshporylation by Cyclin E-Cdk2-P |
| v[59][t] = k_59_*k_112_*m[27][t]*m[40[t] | Cki phosphorylation by Cyclin E-Cdk2-P |
| v[63][t] = k_63_*k_106_*m[38][t]*m[43][t] | Cdk2 phosphorylation by Wee1 |
| v[66][t] = k_66_*k_107_*m[42][t]*(m[44][t]+m[9][t]) | Cdk2 phosphorylation by CAK |
| v[69][t] = k_69_*k_108_*m[30][t]*m[27][t] | Cdc25A phosphorylation by Cyclin E-Cdk2-P |
| v[71][t] = k_71_*k_110_*m[31][t]*m[27][t] | S phase activator phosphorylation by Cyclin E-Cdk2-P |
| v[72][t] = k_72_*k_111_*m[27][t]*m[11][t] | Cki phosphorylation by Cyclin E-Cdk2-P |
| v[86][t] = k_86_*k_117_*m[12][t]*m[19][t] | Rb partial phosphorylation by Cyclin D-Cdk4/6 |
|  |  |
|  | **De-phosphorylation reactions** |
| v[32][t] = k_32_*k_115_*m[20][t]*m[21][t] | Rb de-phosphorylation by PP1 |
| v[37][t] = k_37_*k_116_*m[23][t]*m[24][t] | Rb de-phosphorylation by PP2A |
| v[41][t] = k_41_*k_109_*m[45][t]*m[46][t] | Cdk2 de-phosphorylation by Cdc25A-P |
|  |  |
|  | **Other reactions** |
| v[43][t] = k_43_*m[26][t] | Cyclin E transcriptional activation by E2F |
| v[45][t] = k_45_*m[26][t] | Cdc25A transcriptional activation by E2F |
| v[51][t] = k_51_*m[32][t] | Cyclin E translational activation by Cyclin E mRNA |
| v[82][t] = k_82_*m[26][t] | E2F auto-synthesis |

(b) Ordinary Differential Equations

| **ODE System** |
| --- |
| vol'[t] = k_114_*vol[t] |
| m[1]'[t] = v[1][t] – v[2][t] – v[5][t] – vol'[t]/vol[t]*m[1][t] + vol'[t]/vol[t]*m[1][t] |
| m[2]'[t] = v[3][t] – v[4][t] – v[5][t] – vol'[t]/vol[t]*m[2][t] + vol'[t]/vol[t]*m[2][t] |
| m[3]'[t] = v[5][t] – v[8][t] – v[9][t] – vol'[t]/vol[t]*m[3][t] + vol'[t]/vol[t]*m[3][t] |
| m[4]'[t] = v[6][t] – v[7][t] – v[8][t] – v[11][t] – vol'[t]/vol[t]*m[4][t] + vol'[t]/vol[t]*m[4][t] |
| m[5]'[t] = v[8][t] – v[10][t] – vol'[t]/vol[t]*m[5][t] + vol'[t]/vol[t]*m[5][t] |
| m[6]'[t] = v[9][t]/k_113_ + v[44][t] – v[15][t] |
| m[7]'[t] = v[10][t]/k_113_ – v[44][t] |
| m[8]'[t] = v[11][t]/k_113_ + v[44][t] – v[12][t] – v[78][t] – v[87][t] |
| m[9]'[t] = v[13][t] – v[14][t] |
| m[10]'[t] = v[12][t] – v[16][t] |
| m[11]'[t] = v[16][t] – v[72][t] |
| m[12]'[t] = v[16][t] + v[15][t] – v[20][t] – v[12][t] |
| m[13]'[t] = v[18][t] – v[19][t] |
| m[14]'[t] = v[20][t] – v[21][t] |
| m[15]'[t] = v[21][t] – v[22][t] |
| m[16]'[t] = v[21][t] – v[23][t] |
| m[17]'[t] = v[24][t] – v[25][t] – v[28][t] |
| m[18]'[t] = v[26][t] + v[80][t] + v[81][t] – v[27][t] – v[28][t] – v[79][t] – v[80][t] – v[85][t] |
| m[19]'[t] = v[28][t] + v[32][t] – v[29][t] – v[86][t] |
| m[20]'[t] = v[30][t] – v[31][t] |
| m[21]'[t] = v[29][t] + v[37][t] + v[86][t] – v[32][t] – v[40][t] – v[33][t] |
| m[22]'[t] = v[33][t] – v[34][t] |
| m[23]'[t] = v[35][t] – v[36][t] |
| m[24]'[t] = v[40][t] – v[37][t] – v[38][t] |
| m[25]'[t] = v[38][t] – v[39][t] |
| m[26]'[t] = v[38][t] + v[33][t] + v[82][t] – v [42][t] |
| m[27]'[t] = v[41][t] – v[73][t] – v[87][t] |
| m[28]'[t] = v[43][t] + v[47][t] + v[79][t] – v[49][t] |
| m[30]'[t] = v[45][t] + v[67][t] – v[68][t] – v[69][t] |
| m[31]'[t] = v[46][t] – v[50][t] – v[71][t] |
| m[32]'[t] = v[49][t]*k_113_ – v[51][t] – vol'[t]/vol[t]*m[32][t] + vol'[t]/vol[t]*m[32][t] |
| m[34]'[t] = v[51][t] – v[53][t] – v[55][t] – vol'[t]/vol[t]*m[34][t] + vol'[t]/vol[t]*m[34][t] |
| m[35]'[t] = v[48][t] – v[54][t] – v[55][t] – vol'[t]/vol[t]*m[35][t] + vol'[t]/vol[t]*m[35][t] |
| m[36]'[t] = v[55][t] – v[56][t] – v[76][t] – vol'[t]/vol[t]*m[36][t] + vol'[t]/vol[t]*m[36][t] |
| m[37]'[t] = v[76][t] – v[77][t] – vol'[t]/vol[t]*m[37][t] + vol'[t]/vol[t]*m[37][t] |
| m[38]'[t] = v[56][t]/k_113_ + v[58][t] – v[63][t] |
| m[39]'[t] = v[77][t]/k_113_ – v[58][t] |
| m[40]'[t] = v[58][t] – v[59][t] |
| m[41]'[t] = v[59][t] – v[60][t] |
| m[42]'[t] = v[63][t] – v[66][t] |
| m[43]'[t] = v[61][t] – v[62][t] |
| m[44]'[t] = v[64][t] – v[65][t] |
| m[45]'[t] = v[66][t] – v[41][t] |
| m[46]'[t] = v[69][t] – v[70][t] |
| m[47]'[t] = v[71][t] |
| m[48]'[t] = v[73][t] – v[74][t] |
| m[49]'[t] = v[73][t] – v[75][t] |
| m[50]'[t] = v[72][t] – v[17][t] |
| m[51]'[t] = v[83][t] – v[84][t] |
| m[52]'[t] = v[87][t] |
