# Supplementary material for: Towards a systems biology approach to mammalian cell cycle: modeling the entrance into S phase of quiescent fibroblasts after serum stimulation
Source: BMC Bioinformatics. 2009 Oct 15;10(Suppl 12):S16. doi: 10.1186/1471-2105-10-S12-S16 (PMC2762065; doi:10.1186/1471-2105-10-S12-S16)
Supplement: Additional file 2 — Rate constants in the wild type condition. In bold are the parameters altered in the off state (in parentheses are reported the values for the off state). [file 1471-2105-10-S12-S16-S2.doc]

**Additional file 2**. Rate constants in the wild type condition. In bold are the parameters altered in the *off state* (in parentheses are reported the values for the *off state*).

| **Process** | **Rate constants (WT)** |
| --- | --- |
| **Protein synthesis** (pM*h^-1^) | |
|  | k_1_ = 0.1 |
|  | **k_3_ = 0.45** (0) |
|  | **k_6_ = 0** (1) |
|  | k_13_ = 1 |
|  | k_18_ = 2.5 |
|  | k_24_ = 0 |
|  | k_26_ = 0 |
|  | k_30_ = 0 |
|  | k_35_ = 0 |
|  | k_46_ = 1 |
|  | k_47_ = 0.0035 |
|  | k_48_ = 0.1 |
|  | k_61_ = 1 |
|  | k_64_ = 2 |
|  | k_67_ = 0.1 |
|  | **k_83_ = 0.37** (0) |
| **Protein degradation** (h^-1^) | |
|  | k_2_ = 0 |
|  | **k_4_ = 3.5** (35) |
|  | k_7_ = 0 |
|  | k_14_ = 0.1 |
|  | k_17_ = 5 |
|  | k_19_ = 0.01 |
|  | k_22_ = 10 |
|  | k_23_ = 10 |
|  | k_25_ = 0 |
|  | k_27_ = 0 |
|  | k_31_ = 0 |
|  | k_34_ = 10 |
|  | k_36_ = 0 |
|  | k_39_ = 0.1 |
|  | k_42_ = 0.6 |
|  | k_50_ = 0.05 |
|  | k_53_ = 0.05 |
|  | k_54_ = 0.3 |
|  | k_60_ = 5 |
|  | k_62_ = 0.5 |
|  | k_65_ = 0 |
|  | k_68_ = 0 |
|  | k_70_ = 0.01 |
|  | k_74_ = 10 |
|  | k_75_ = 10 |
|  | k_84_ = 0.2 |
| **Complex formation** (pM^-1^*h^-1^) | |
|  | k_5_ = 0.5 |
|  | k_8_ = 1 |
|  | k_12_ = 0.001 |
|  | k_28_ = 0 |
|  | k_55_ = 2 |
|  | k_76_ = 0.1 |
|  | k_87_ = 0.1 |
| **Complex dissociation** (h^-1^) | |
|  | k_16_ = 0.1 |
|  | k_21_ = 0.1 |
|  | k_33_ = 1 |
|  | k_38_ = 3 |
|  | k_44_ = 0.5 |
|  | k_53_ = 0.05 |
|  | k_73_ = 0.01 |
| **Translocation** (h^-1^) | |
|  | k_9_ = 0.25 |
|  | k_10_ = 1.25 |
|  | k_11_ = 0.25 |
|  | k_49_ = 0.5 |
|  | k_56_ = 0.25 |
|  | k_77_ = 1.25 |
|  | k_78_ = 0.25 |
| **Phosphorylation**  **(km and kcat)** (pM) |  |
|  | k_15_ = 1 |
|  | k_20_ = 0.5 |
|  | k_29_ = 0 |
|  | k_40_ = 2 |
|  | k_59_ = 5 |
|  | k_63_ = 1 |
|  | k_66_ = 2 |
|  | k_69_ = 2.5 |
|  | k_71_ = 1.5 |
|  | k_72_ = 5 |
|  | k_86_ = 1 |
|  | k_100_ = 0.1 |
|  | k_101_ = 0.01 |
|  | k_102_ = 0 |
|  | k_103_ = 1 |
|  | k_106_ = 0.3 |
|  | k_107_ = 0.3 |
|  | k_108_ = 1 |
|  | k_110_ = 0.1 |
|  | k_111_ = 5 |
|  | k_112_ = 5 |
|  | k_117_ = 5 |
| **De-phosphorylation**  **(km and kcat)** (pM) |  |
|  | k_32_ = 0 |
|  | k_37_ = 0 |
|  | k_41_ = 3 |
|  | k_109_ = 1 |
|  | k_115_ = 0 |
|  | k_116_ = 0 |
| **Other reactions** (pM^-1^*h^-1^) |  |
|  | k_43_ = 2 |
|  | k_45_ = 2 |
|  | k_51_ = 5 |
|  | k_82_ = 1 |
| **Growth** |  |
|  | k_113_ = V_nucleus_/V_cytoplasm_ |
|  | k_114_ = 0.028 |
