# Supplementary material for: Towards a systems biology approach to mammalian cell cycle: modeling the entrance into S phase of quiescent fibroblasts after serum stimulation
Source: BMC Bioinformatics. 2009 Oct 15;10(Suppl 12):S16. doi: 10.1186/1471-2105-10-S12-S16 (PMC2762065; doi:10.1186/1471-2105-10-S12-S16)
Supplement: Additional file 3 — Initial concentration values in both wild type and off state. [file 1471-2105-10-S12-S16-S3.doc]

**Additional file 3.** Initial concentration values in both wild type and *off state*.

| **Component** | **Concentration value (t=0)** pM |
| --- | --- |
| **Cyclins** |  |
| Cyclin D cytoplasm | m[2] = 0.02 |
| Cyclin D-P nucleus | m[16] = 0 |
| Cyclin E mRNA nucleus | m[28] = 0 |
| Cyclin E mRNA cytoplasm | m[32] = 0 |
| Cyclin E cytoplasm | m[34] = 0.005 |
| Cyclin E nucleus | m[49] = 0 |
| **Cdks** |  |
| Cdk4/6 cytoplasm | m[1] = 0.88 |
| Cdk4/6-P nucleus | m[15] = 0 |
| Cdk2 cytoplasm | m[35] = 2 |
| Cdk2-P nucleus | m[48] = 0 |
| **Cdk-Inhibitors** |  |
| Cki cytoplasm | m[4] = 10 |
| Cki nucleus | m[8] = 0 |
| Cki nucleus from Cyclin D-Cdk4/6-P-Cki | m[11] = 0 |
| Cki nucleus from Cyclin E-Cdk2-Cki | m[40] = 0 |
| Cki-P nucleus | m[41] = 0 |
| Cki-P nucleus | m[50] = 0 |
| **Transcription factors** |  |
| E2F free nucleus | m[18] = 0 |
| E2F active nucleus | m[26] = 0 |
| **Retinoblastoma proteins** |  |
| Rb free nucleus | m[17] = 0 |
| Rb-P nucleus | m[22] = 0 |
| Rb-P-P nucleus | m[25] = 0 |
| **Complexes** |  |
| Cyclin D-Cdk4/6 cytoplasm | m[3] = 0 |
| Cyclin D-Cdk4/6-Cki cytoplasm | m[5] = 0 |
| Cyclin D-Cdk4/6 nucleus | m[6] = 0 |
| Cyclin D-Cdk4/6-Cki nucleus | m[7] = 0 |
| Cyclin D-Cdk4/6-P-Cki nucleus | m[10] = 0 |
| Cyclin D-Cdk4/6-P nucleus | m[12] = 0 |
| Cyclin D-P-Cdk4/6-P nucleus | m[14] = 0 |
| E2F-Rb nucleus | m[19] = 0.1 |
| E2F-Rb-P nucleus | m[21] = 0 |
| E2F-Rb-P-P nucleus | m[24] = 0 |
| Cyclin E-Cdk2-P nucleus | m[27] = 0 |
| Cyclin E-Cdk2 cytoplasm | m[36] = 0 |
| Cyclin E-Cdk2-Cki cytoplasm | m[37] = 0 |
| Cyclin E-Cdk2 nucleus | m[38] = 0 |
| Cyclin E-Cdk2-Cki nucleus | m[39] = 0 |
| Cyclin E-Cdk2-P nucleus | m[42] = 0 |
| Cyclin E-Cdk2-P-P-P nucleus | m[45] = 0 |
| Cyclin E-Cdk2-P-Cki nucleus | m[52] = 0 |
| **Other Kinases** |  |
| CAK nucleus on Cyclin D-Cdk4/6 | m[9] = 0 |
| GSK3beta nucleus | m[13] = 0.4 |
| Wee1 nucleus | m[43] = 0 |
| CAK nucleus on Cyclin E-Cdk2 | m[44] = 0 |
| **Phosphatases** |  |
| PP1 nucleus | m[20] = 0 |
| PP2A nucleus | m[23] = 0 |
| Cdc25A nucleus | m[30] = 0 |
| Cdc25A-P nucleus | m[46] = 0.02 |
| **S phase activator** |  |
| Cdc6 nucleus | m[31] = 0.001 |
| Cdc6-P nucleus | m[47] = 0 |
| **Synthesis activation factor** |  |
| Generic serum-dependent modifier | m[51] = 0.08 |
| **Volume** |  |
| Initial volume cytoplasm | Volume = 2.308*10^-11^ |
